# Dental care and oral conditions are associated with the prevalence of sarcopenia in people with type 2 diabetes: a cross-sectional study

**DOI:** 10.1186/s12902-023-01331-4

**Published:** 2023-04-07

**Authors:** Fuyuko Takahashi, Yoshitaka Hashimoto, Hiroshi Okada, Yuriko Kondo, Ayumi Kaji, Ryosuke Sakai, Yuka Kawate, Takuro Okamura, Naoko Nakanishi, Saori Majima, Takafumi Senmaru, Emi Ushigome, Masahide Hamaguchi, Mai Asano, Masahiro Yamazaki, Michiaki Fukui

**Affiliations:** 1 Department of Endocrinology and Metabolism, Graduate School of Medical Science, Kyoto Prefectural University of Medicine, 465, Kajii cho, Kamigyo-ku, Kyoto-city, Kyoto 602-8566 Japan; 2 Department of Diabetes and Endocrinology, Matsushita Memorial Hospital, Moriguchi, 570- 8540 Japan

**Keywords:** Dental care, Oral conditions, sarcopenia, Type 2 diabetes mellitus

## Abstract

**Background:**

Insulin resistance, which is closely associated with type 2 diabetes mellitus (T2DM), is a cause of sarcopenia and people with T2DM have a high risk of sarcopenia. Keeping good oral condition by dental care is important for people with T2DM. Keeping good oral condition by dental care is important for people with T2DM. This study has investigated the association between dental care or oral conditions and sarcopenia in people with T2DM.

**Methods:**

Dental care and oral conditions were evaluated based on a self-reported questionnaire. Individuals with both low handgrip strength and low skeletal muscle mass index were diagnosed with sarcopenia.

**Results:**

Among 266 people with T2DM, the proportions of sarcopenia, not having a family dentist, not having a toothbrushing behavior, poor chewing ability, and use of complete dentures were 18.0%, 30.5%, 33.1%, 25.2%, and 14.3%, respectively. The proportions of sarcopenia in people not having a family dentist (27.2% vs. 14.1%, *p* = 0.017), those with poor chewing ability (26.9% vs. 15.1%, *p* = 0.047), and use of complete dentures (36.8% vs. 14.9%, *p* = 0.002) were higher than those in people without. The proportion of sarcopenia in people without toothbrushing behavior tended to be higher than that in people with toothbrushing behavior (25.0% vs. 14.6%, *p* = 0.057). Not having a family dentist (adjusted odds ratio [OR] 2.48 [95% confidence interval (CI): 1.21–5.09], *p* = 0.013), poor chewing ability (adjusted OR 2.12 [95% CI: 1.01–4.46], *p* = 0.048), and use of complete dentures (adjusted OR 2.38 [95% CI: 1.01–5.99], *p* = 0.046) were related to the prevalence of sarcopenia.

**Conclusions:**

This study revealed that dental care and oral conditions were associated with the prevalence of sarcopenia.

## Background

The population of people with type 2 diabetes mellitus (T2DM) is increasing worldwide [1]. T2DM is a chronic disease characterized by hyperglycemia because of insulin resistance (IR). In IR states, insulin-stimulated glucose disposal is severely impaired in the skeletal muscle [2]. Therefore, IR induces loss of muscle mass. Sarcopenia, which is defined as muscle strength, mass, and function loss [3] with age, has been associated with cardiovascular disease (CVD) [4] and low quality of life [5]. In addition, sarcopenia is known as a risk factor for mortality [6, 7]. People with T2DM have been reported to have a 1.55-to-3-fold higher risk of sarcopenia than the general population, since people with T2DM recognize IR [8, 9]. Therefore, sarcopenia in people with T2DM requires more attention than that in individuals without diabetes.

People with T2DM had a higher risk of periodontal disease than those without [10]. The severity of periodontal disease was related to glucose tolerance status and the development of glucose intolerance [11] and glycosylated hemoglobin (HbA1c) levels [12, 13]. Furthermore, the severity of periodontal disease affects inflammation and IR [14]. Infection with *porphyromonas gingivalis*, which causes periodontal disease, is a risk of metabolic syndrome and skeletal muscle metabolic dysfunction via gut microbiome alteration [15]. Furthermore, toothbrushing behavior was associated with smaller increments in the number of teeth with periodontal pocketing [16]. Therefore, it is important for people with diabetes to have a family dentist and regular visits with their dentist.

Chewing is a process that includes predation, crushing, and mixing of food; the formation of a bolus; and delivery of that bolus to the pharynx, which greatly affects food intake [17]. Chewing ability has been shown to be associated with sarcopenia in the general population [18]. In addition, several studies have reported the relationship between chewing ability and muscle strength [19], physical performance [20] and all-cause mortality [21]. Moreover, we also reported that low tongue pressure was related to the presence of sarcopenia [22]. On the other hand, poor chewing ability was associated with the use of removable dentures [23]. The use of complete dentures has been shown to be related to the presence of low handgrip strength [24]. However, previous studies have not researched the relationship between dental care and oral conditions, such as having a family dentist, toothbrushing behavior, chewing ability or use of complete dentures, and the presence of sarcopenia in people with T2DM. Therefore, this cross-sectional study researched the association between dental care and oral conditions, such as having a family dentist, toothbrushing, chewing ability, or use of complete dentures, and sarcopenia in people with T2DM.

## Methods

### Study design, setting, and participants

The KAMOGAWA-DM cohort study, which is a cohort study in progress with diabetes mellitus, was introduced in 2014 to understand the natural disease history of individuals with diabetes mellitus [25]. The KAMOGAWA-DM cohort study included outpatients at the Department of Endocrinology and Metabolism, Kyoto Prefectural University of Medicine Hospital (Kyoto, Japan). The present study was approved by the Research Ethics Committee of Kyoto Prefectural University of Medicine (No. RBMR-E-466-6) and was conducted in accordance with the principles of the Declaration of Helsinki. After obtaining written informed consent, medical data were anonymously collected and compiled into a database. This study included people with T2DM who responded to questionnaires about dental care and oral conditions from March 2015 to April 2021 and agreed to participate in the KAMOGAWA-DM cohort study. The exclusion criteria were as follows: 1) no data on body composition and 2) no data on handgrip strength.

### Questionnaire about lifestyle characteristics and chewing ability

Family history of diabetes, duration of diabetes, smoking status, exercise habit, and alcohol consumption habit were assessed using a standardized questionnaire. Based on their responses to the questionnaire, “exercise habit” was defined as carrying out any type of physical activity once or more per week, “smoking habit” was defined as smoking cigarettes or another tobacco product currently, and “alcohol consumption habit” was defined as daily alcohol consumption.

### Dental care and oral condition questionnaire

Participants were grouped into two groups: those who had a family dentist or those who did not have a family dentist. The frequency of toothbrushing was how often they brushed their teeth per day: none, sometimes, once, twice, thrice, four times, or five times or more per day. We defined people with toothbrushing behavior if they brushed their teeth ≥ twice per day [16]. Chewing ability was evaluated by the following statements: “I can chew and eat anything,” “There are some food I cannot chew,” “There are many food I cannot chew,” or “I cannot eat with chewing.” In this study, “I can chew and eat anything” was defined as good chewing ability and “There are some food I cannot chew,” “There are many food I cannot chew,” or “I cannot eat with chewing” were defined as having poor chewing ability [26]. Participants were grouped into two groups: those with or without complete denture usage.

### Participants’ data

After fasting overnight, venous blood samples were collected to measure the concentrations of fasting plasma glucose, high-density lipoprotein cholesterol, triglycerides, uric acid, and creatinine. Glycosylated hemoglobin (HbA1c) was measured by high-performance liquid chromatography and expressed in the National Glycohemoglobin Standardization Program. The estimated glomerular filtration rate (eGFR; mL/min/1.73 m^2^) was estimated as follows: eGFR = 194 × serum creatinine^− 1.094^× age^− 0.287^ (×0.739 for women) [27]. Blood pressure measurements were performed automatically using an automatic blood pressure measurement device (HEM-906; OMRON, Kyoto, Japan) after resting for 5 min in a quiet room. The handgrip strength of each hand was tested by a handgrip dynamometer (Smedley, Takei Scientific Instruments Co., Ltd., Niigata, Japan) twice with each hand, and the maximum value was recorded and used for analysis.

Body composition was assessed using a multifrequency impedance body composition analyzer, InBody 720 (InBody Japan, Tokyo, Japan), which has been shown to have good correlation with dual-energy X-ray absorptiometry [28]. Using this analyzer, body weight (BW, kg) and appendicular muscle mass (kg) were determined, and then, body mass index (BMI, kg/m^2^) and skeletal muscle mass index (SMI, kg/m^2^) were calculated, BMI = BW (kg)/height squared (m^2^) and SMI = appendicular muscle mass (kg)/ height squared (m^2^), respectively.

Data of medications for diabetes, including glucagon-like peptide-1 agonist, insulin, sodium glucose cotransporter-2 inhibitor, metformin, dipeptidyl peptide 4 inhibitor, sulfonylurea, thiazolidines, glinides, α-glycosidase inhibitors and antihypertensive drugs, were obtained from medical records.

Having hypertension was defined as antihypertensive drugs usage, systolic blood pressure ≥ 140 mmHg, and/or diastolic blood pressure ≥ 90 mmHg.

### Definition of sarcopenia

Sarcopenia was defined according to the Asian Working Group for Sarcopenia guidelines, utilizing SMI and handgrip strength [3]. People who had both low muscle strength, indicating handgrip strength < 28 kg for men and < 18 kg for women, and low skeletal muscle mass indicating SMI < 7.0 kg/m^2^ for men and < 5.7 kg/m^2^ for women, were diagnosed with sarcopenia [3].

### Statistical analyses

Data are presented as frequencies of potential confounding variables or means (standard deviation [SD]). The participants were classified into the following two groups based on having a family dentist, toothbrushing behavior, chewing ability and use of complete dentures. The differences in the continuous variables and categorical variables were evaluated using Student’s t-test and chi-square test, respectively. Logistic regression analyses were run to determine the odds ratio (OR) and 95% confidence interval (CI) for having a family dentist, toothbrushing behavior, chewing ability, or use of complete dentures in the presence of sarcopenia, adjusting for age, sex, smoking habits and exercise habits.

Statistical analyses were conducted using EZR (Saitama Medical Center, Jichi Medical University, Saitama, Japan) [29], a graphical user interface for R (The R Foundation for Statistical Computing, Vienna, Austria). Differences were considered statistically significant at *p* values of < 0.05.

## Results

**Fig. 1:**
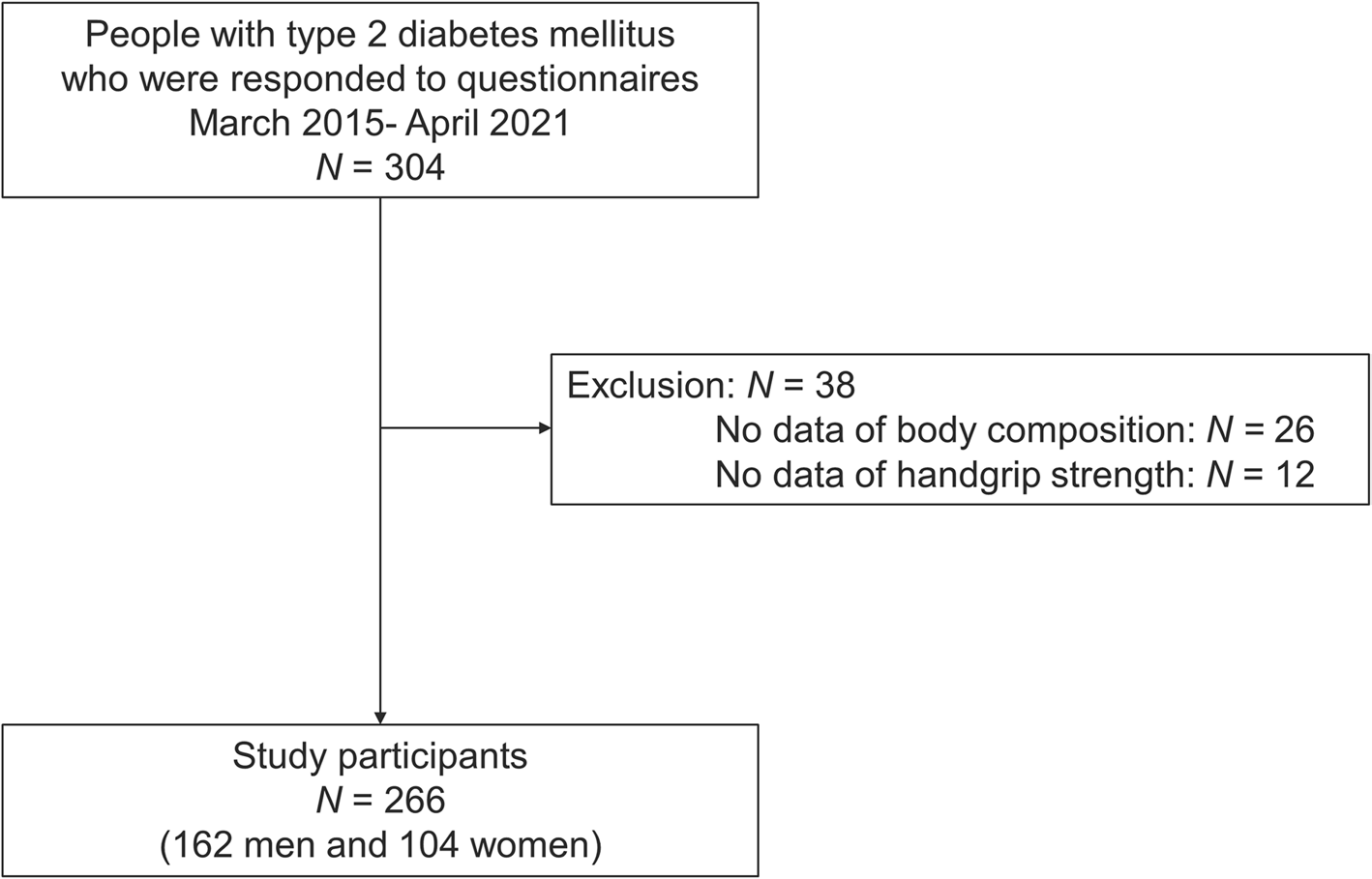
Study flow diagram for the registration of participants

A total of 304 individuals with T2DM were included in the present study. We excluded 38 people: 26 who did not undergo the multifrequency impedance body composition analyzer test, and 12 who did not undergo measurement of handgrip strength; finally, a total of 266 people (162 men and 104 women) were included in this study (shown in Fig. 1).

The clinical characteristics of the study participants are summarized in Table 1. Mean age, BMI, SMI, and handgrip strength were 69.1 ± 8.7 years, 23.6 ± 3.9 kg/m^2^, 6.9 ± 1.1 kg/m^2^, and 26.4 ± 8.5 kg, respectively. The proportion of sarcopenia was 18.0% (*n* = 48), and the proportions of participants not having a family dentist, those without a toothbrushing behavior, those with poor chewing ability, and those with complete dentures usage were 30.5% (*n* = 81), 33.1% (*n* = 88), 25.2% (*n* = 67), and 14.3% (*n* = 38), respectively. Metformin and dipeptidyl peptide 4 inhibitor were used 43.6% (*n* = 116) and 35.3% (*n* = 94).

**Table 1:** Clinical characteristics of study participants

	All *N* = 266
Age (years)	69.1 (8.7)
Sex (men/women)	162/104
Duration of diabetes (years)	17.6 (11.6)
Family history of diabetes (-/+)	154/112
BMI (kg/m^2^)	23.6 (3.9)
SBP (mmHg)	130.7 (16.1)
DBP (mmHg)	73.8 (11.6)
Antihypertensive drugs (-/+)	108/158
Presence of hypertension (-/+)	89/177
Insulin (-/+)	196/70
SGLT2 inhibitor (-/+)	212/54
GLP-1 agonist (-/+)	234/32
DPP4 inhibitor (-/+)	94/172
Metformin (-/+)	150/116
SU (-/+)	177/89
Thiazolidine (-/+)	253/13
Glinide (-/+)	228/38
α-glycosidase inhibitor (-/+)	229/37
Habit of smoking (-/+)	227/39
Habit of exercise (-/+)	124/142
Habit of drinking alcohol (-/+)	188/78
HbA1c (mmol/mol)	56.6 (10.6)
HbA1c (%)	7.3 (1.0)
Plasma glucose (mmol/L)	8.1 (2.2)
Creatinine (umol/L)	74.1 (33.5)
SMI (kg/m^2^)	6.9 (1.1)
Low skeletal muscle mass (-/+)	183/83
Handgrip strength (kg)	26.4 (8.5)
Low muscle strength (-/+)	178/88
Presence of sarcopenia (-/+)	218/48
Family dentist (-/+)	81/185
Toothbrushing behavior (-/+)	88/178
Chewing ability (good/poor)	199/67
Use of complete dentures (-/+)	228/38

Data was expressed as mean (standard deviation) or number

*BMI* Body mass index, *SBP* Systolic blood pressure, *DBP* Diastolic blood pressure, *SGLT2* Sodium glucose cotransporter-2, *GLP-1* Glucagon-like peptide-1, *DPP4* Dipeptidyl peptide 4, *SU* Sulfonylurea, *SMI* Skeletal muscle mass index

Table 2 reveals the results of the clinical characteristics of the participants according to dental care and oral condition. The proportion of sarcopenia with people not having a family dentist was higher than those having a family dentist (27.2% vs. 14.1%, *p* = 0.017). The proportion of sarcopenia with poor chewing ability was higher than those with good chewing ability (26.9% vs. 15.1%, *p* = 0.047), and those with use of complete denture were higher than those without use of complete denture (36.8% vs. 14.9%, *p* = 0.002). The proportion of sarcopenia in people without toothbrushing behavior tended to be higher than that in people with toothbrushing behavior, although the difference was not statistically significant (25.0% vs. 14.6%, *p* = 0.057). The proportion of not having a family dentist (52.6% vs. 26.8%, *p* = 0.003), no toothbrushing behavior (55.3% vs. 20.2%, *p* < 0.001), and low chewing ability (55.3% vs. 20.2%, *p* < 0.001) in people with use of complete dentures was higher than those without.

**Table 2:** Clinical characteristics according to dental care and oral conditions

	Family dentist			Toothbrushing behavior			Chewing ability			Use of complete dentures		
	(-) *N* = 81	(+) *N* = 185	*p*	(-) *N* = 88	(+) *N* = 178	*p*	(Poor) *N* = 67	(Good) *N* = 199	*p*	(-) *N* = 228	(+) *N* = 38	*p*
Age (years)	69.6 (9.1)	68.9 (8.5)	0.515	70.2 (9.3)	68.6 (8.3)	0.147	70.5 (7.1)	69.7 (9.1)	0.138	68.2 (8.7)	74.4 (6.5)	**< 0.001**
Sex (men/women)	55/26	107/78	0.158	68/20	94/84	**< 0.001**	40/27	122/77	0.930	133/95	29/9	0.054
Duration of diabetes (years)	18.0 (11.4)	17.4 (11.7)	0.732	19.2 (12.2)	16.8 (11.3)	0.114	20.0 (12.0)	16.8 (11.4)	0.054	17.2 (11.5)	20.3 (12.3)	0.120
Family history of diabetes (-/+)	51/30	103/82	0.331	53/35	101/77	0.682	41/26	113/86	0.625	128/100	26/12	0.214
BMI (kg/m^2^)	23.0 (3.3)	23.8 (4.1)	0.098	23.8 (4.4)	23.4 (3.7)	0.498	22.9 (3.6)	23.8 (4.0)	0.101	23.7 (3.9)	22.8 (3.8)	0.219
SBP (mmHg)	129.5 (17.7)	131.2 (15.4)	0.425	134.4 (16.2)	128.8 (15.8)	**0.008**	128.9 (16.9)	131.3 (15.8)	0.296	129.7 (15.3)	136.2 (19.7)	**0.022**
DBP (mmHg)	73.1 (13.0)	74.1 (10.9)	0.529	75.4 (12.2)	72.9 (11.2)	0.096	71.4 (12.2)	74.6 (11.3)	0.055	73.7 (11.2)	74.3 (13.6)	0.763
Antihypertensive drugs (-/+)	31/50	77,108	0.707	24/64	84/94	**0.003**	26/41	82/117	0.840	100/128	8/30	**0.013**
Presence of hypertension (-/+)	29/52	60/125	0.693	21/67	68/110	**0.028**	19/48	70/129	0.383	83/145	6/32	**0.021**
Insulin (-/+)	61/20	135/50	0.805	62/26	134/44	0.488	41/26	155/44	**0.012**	171/57	25/13	0.320
SGLT2 inhibitor (-/+)	65/16	147/38	1.000	66/22	146/32	0.239	50/17	162/37	0.309	180/48	32/6	0.597
GLP-1 agonist (-/+)	76/5	158/27	0.082	70/18	164/14	**0.006**	57/10	177/22	0.532	201/27	33/5	1.000
Habit of smoking (-/+)	67/14	160/25	0.541	69/19	158/20	**0.039**	52/15	175/24	0.062	194/34	33/5	0.972
Habit of exercise (-/+)	40/41	84/101	0.642	48/40	76/102	0.091	30/37	94/105	0.836	106/122	18/20	1.000
Habit of drinking alcohol (-/+)	18/63	125/60	0.124	54/34	134/44	**0.028**	46/21	142/57	0.791	162/66	26/12	0.891
HbA1c (mmol/mol)	56.7 (11.8)	56.5 (10.1)	0.931	58.3 (12.0)	55.7 (9.7)	0.061	58.1 (13.7)	56.1 (9.3)	0.172	56.6 (10.4)	56.3 (11.9)	0.889
HbA1c (%)	7.3 (1.1)	7.3 (0.9)	0.931	7.5 (1.1)	7.2 (0.9)	0.061	7.5 (1.3)	7.3 (0.9)	0.172	7.3 (1.0)	7.3 (1.1)	0.889
Plasma glucose (mmol/L)	8.1 (2.1)	8.0 (2.2)	0.841	8.5 (2.4)	7.8 (2.0)	**0.010**	7.9 (2.0)	8.1 (2.2)	0.438	8.0 (2.1)	8.7 (2.7)	0.054
Creatinine (umol/L)	79.9 (47.8)	71.5 (24.6)	0.061	80.3 (42.7)	71.0 (27.6)	0.032	70.9 (24.2)	75.1 (36.1)	0.376	73.1 (34.6)	80.1 (25.5)	0.234
SMI (kg/m^2^)	6.8 (1.0)	6.9 (1.1)	0.335	7.1 (1.1)	6.8 (1.1)	**0.028**	6.7 (1.1)	6.9 (1.1)	0.116	6.9 (1.1)	6.7 (1.1)	0.441
Low skeletal muscle mass (-/+)	49/32	134/51	0.073	61/27	122/56	1.000	36/31	147/52	**0.003**	163/65	20/18	**0.033**
Handgrip strength (kg)	25.5 (8.6)	26.8 (8.5)	0.282	27.3 (9.4)	25.9 (8.1)	0.205	24.9 (7.8)	26.9 (8.7)	0.104	26.7 (8.6)	24.4 (7.9)	0.114
Low muscle strength (-/+)	46/35	132/53	**0.029**	47/41	131/47	**0.002**	40/27	138/61	0.193	163/65	15/23	**< 0.001**
Presence of sarcopenia (-/+)	59/22	159/26	**0.017**	66/22	152/26	0.057	49/18	169/30	**0.047**	194/34	24/14	**0.002**
Family dentist (-/+)	-	-	-	32/56	49/129	0.183	26/41	55/144	0.118	61/167	20/18	**0.003**
Toothbrushing behavior (-/+)	32/49	56/129	0.183	-	-	-	21/46	67/132	0.842	66/162	22/16	**< 0.001**
Chewing ability (poor/good)	26/55	41/144	0.118	21/67	46/132	0.842	-	-	-	46/182	21/17	**< 0.001**
Use of complete dentures (-/+)	61/20	167/18	**0.003**	66/22	162/16	**< 0.001**	46/21	182/17	**< 0.001**	-	-	-

Data was expressed as mean (standard deviation) or number. The difference between group was evaluated by Student’s t-test, or chi-square test

*BMI* Body mass index, *SBP* Systolic blood pressure, *DBP* Diastolic blood pressure, *SGLT2* Sodium glucose cotransporter-2, *GLP-1* Glucagon-like peptide-1, *SMI* Skeletal muscle mass index

Furthermore, not having a family dentist (adjusted OR, 2.48 [95% CI: 1.21–5.09], *p* = 0.013), poor chewing ability (adjusted OR, 2.12 [95% CI: 1.01–4.46], *p* = 0.048), and use of complete dentures (adjusted OR, 2.38 [95% CI: 1.01–5.99], *p* = 0.046) were related to the presence of sarcopenia. The absence of toothbrushing behavior was associated with the presence of sarcopenia (unadjusted OR, 1.95 [95% CI: 1.03–3.68], *p* = 0.040), although it was not statistically significant after adjusting for covariates (adjusted OR, 1.71 [95% CI: 0.81–3.59], *p* = 0.157) (Table 3).

**Table 3:** Odds ratio of dental care and oral conditions on the presence of sarcopenia

	Model 1		Model 2	
	OR (95%CI)	*p*	OR (95% CI)	*p*
Family dentist (-)	2.28 (1.20–4.33)	**0.001**	2.48 (1.21–5.09)	**0.013**
Age (years)	-	-	1.15 (1.09–1.22)	**< 0.001**
Women	-	-	1.86 (0.89–3.86)	0.098
Habit of exercise	-	-	0.44 (0.21–0.88)	**0.021**
Habit of smoking	-	-	1.55 (0.54–4.41)	**0.041**
Toothbrushing behavior (-)	1.95 (1.03–3.68)	**0.040**	1.71 (0.81–3.59)	0.157
Age (years)	-	-	1.14 (1.08–1.21)	**< 0.001**
Women	-	-	1.87 (0.89–3.91)	0.097
Habit of exercise	-	-	0.46 (0.23–0.92)	**0.029**
Habit of smoking	-	-	1.48 (0.52–4.19)	0.461
Chewing ability (poor)	2.07 (1.06–4.03)	**0.032**	2.12 (1.01–4.46)	**0.048**
Age (years)	-	-	1.15 (1.09–1.22)	**< 0.001**
Women	-	-	1.69 (0.82–3.48)	0.154
Habit of exercise	-	-	0.44 (0.22–0.88)	**0.021**
Habit of smoking	-	-	1.38 (0.47–4.05)	0.056
Use of complete dentures (+)	3.33 (1.57–7.07)	**0.002**	2.38 (1.01–5.99)	**0.046**
Age (years)	-	-	1.14 (1.08–1.20)	**< 0.001**
Women	-	-	1.90 (0.91–3.97)	0.087
Habit of exercise	-	-	0.43 (0.21–0.88)	**0.020**
Habit of smoking	-	-	1.67 (0.59–4.71)	0.335

Model 1 is unadjusted; Model 2 is adjusted for age, sex, habit of exercise and habit of smoking

## Discussion

The present study is the first investigation of the relationship between dental care and oral conditions, such as having a family dentist, toothbrushing behavior, chewing ability or usage of complete dentures, and the prevalence of sarcopenia in people with T2DM. The results of the present study showed that not having a family dentist, poor chewing ability, and use of complete dentures were associated with a higher prevalence of sarcopenia.

Possible explanations for the association between dental care or oral condition and a higher prevalence of sarcopenia are as follows:

Periodontal disease severity affects chronic inflammation and IR [14]. Chronic inflammation that occurs in response to many kinds of bacterial community in the subgingival region is features of periodontal disease [30]. Although this chronic inflammatory happens locally in the oral cavity, inflammatory mediators produced by periodontitis, as well as bacteria, can expand from the oral cavity, causing various diseases outside the oral cavity [30]. Inflammatory cytokines, such as tumor necrosis factor-α (TNF-α), can trigger IR states [31], and the epidemiological studies also have reported that inflammation is an independent risk of both IR [32] and T2DM [33, 34]. IR has been shown to be a reason for sarcopenia [35, 36]. Furthermore, periodontal disease is recognized as a risk factor for metabolic dysfunction of skeletal muscle [15]. In this study, the proportion of sarcopenia in people who had a family dentist was lower than that in people who did not have a family dentist. This suggests that having a family dentist and maintaining good oral health may reduce IR and prevent sarcopenia, although the presence or absence of periodontal disease was not evaluated. Furthermore, toothbrushing is considered a prerequisite for maintaining good oral health and preventing periodontal disease [16]. In this study, the proportion of sarcopenia in people without toothbrushing behavior was higher than that in those with toothbrushing behavior, although the results of multivariate analysis were not statistically significant. A previous study showed that toothbrushing behavior was related to handgrip strength [37]. *Porphyromonas gingivalis*, which is periodontitis bacteria, impairs glucose uptake in skeletal muscle associated with altering gut microbiota [15]. In this study, toothbrushing behavior was associated with the presence of low muscle strength. Although further research is needed, toothbrushing may prevent sarcopenia, because toothbrushing protect the development of periodontal disease.

In addition, maintaining good oral health prevents oral frailty. Oral frailty, which is now recognized as the accumulation of a poor oral function and condition, is reported to be associated with risk of incident mortality, malnutrition, dysphagia, physical frailty, and need for long-term care, and oral frailty causes poor chewing ability [38]. Previous studies have reported the relationship between chewing ability and handgrip strength [39] or general function [20]. Furthermore, chewing ability has been found to be related to sarcopenia in the general population [18]. Poor chewing ability has been known to be a risk factor for malnutrition [40]. In this study, the presence of sarcopenia in people with poor chewing ability was higher than those with good chewing ability. Therefore, maintaining good chewing ability may prevent sarcopenia.

A previous study showed that the use of complete dentures is associated with the presence of low handgrip strength [24]. In this study, the use of complete dentures was related to the presence of sarcopenia. People who use complete dentures often have denture stomatitis, which is a common inflammatory disease that affects the mucosa under complete dentures, and the progression of denture stomatitis without treatment may cause systemic infection [41]. Oral infections increase the levels of interleukin-6 and TNF-α receptors [42], which are associated with inflammation.

However, there were certain limitations in this study. First, the data of dental care and oral health status were based on self-reporting, and some concerns were raised the accuracy of the data. Second, the presence or absence of periodontal disease and denture stomatitis were not evaluated. Finally, the design of this study was cross-sectional in nature. Thus, the causal relationship between dental care and oral condition, such as having a family dentist, toothbrushing behavior, chewing ability, or use of complete dentures, and the prevalence of sarcopenia is unclear. Moreover, having a family dentist, toothbrushing behavior, chewing ability, and use of complete dentures may affect each other.

## Conclusions

This study identified that not having a family dentist, poor chewing ability, and use of complete dentures were related to a higher prevalence of sarcopenia in people with T2DM. Clinicians should pay attention to the dental care and oral conditions of individuals with T2DM to prevent sarcopenia.

## Data Availability

The datasets used and/or analysed during the current study are available from the corresponding author on reasonable request.

## Abbreviations

BDHQ: Brief-type self-administered diet history questionnaire
BMI: Body mass index
BW: Body weight
CI: Confidence interval
CVD: Cardiovascular disease
eGFR: Estimated glomerular filtration rate
GLP-1: Glucagon-like peptide-1
HbA1c: Glycosylated hemoglobin
OR: Odds ratio
SD: Standard deviation
SGLT2: Sodium-glucose cotransporter-2
SMI: Skeletal muscle mass index
T2DM: Type 2 diabetes mellitus
